# Activation of P2×7 Receptor Promotes the Invasion and Migration of Colon Cancer Cells via the STAT3 Signaling

**DOI:** 10.3389/fcell.2020.586555

**Published:** 2020-11-24

**Authors:** Wen-jun Zhang, Ce-gui Hu, Hong-liang Luo, Zheng-ming Zhu

**Affiliations:** The Second Affiliated Hospital, Nanchang University, Nanchang, China

**Keywords:** colon cancer, LOVO and SW480 cells, P2 × 7 receptor, invasion and migration, ATP

## Abstract

The pathological mechanism of colon cancer is very complicated. Therefore, exploring the molecular basis of the pathogenesis of colon cancer and finding a new therapeutic target has become an urgent problem to be solved in the treatment of colon cancer. ATP plays an important role in regulating the progression of tumor cells. P2 × 7 belongs to ATP ion channel receptor, which is involved in the progression of tumors. In this study, we explored the effect and molecular mechanism of ATP-mediated P2 × 7 receptor on the migration and metastasis of colon cancer cells. The results showed that ATP and BzATP significantly increased the inward current and intracellular calcium concentration of LOVO and SW480 cells, while the use of antagonists (A438079 and AZD9056) could reverse the above phenomenon. We found that ATP promoted the migration and invasion of LOVO and SW480 cells and is dose-dependent on ATP concentration (100–300 μM). Similarly, BzATP (10, 50, and 100 μM) also significantly promoted the migration and invasion of colon cancer cells in a concentration-dependent manner. While P2 × 7 receptor antagonists [A438079 (10 μM), AZD9056 (10 μM)] or P2 × 7 siRNA could significantly inhibit ATP-induced colon cancer cell migration and invasion. Moreover, *in vivo* experiments showed that ATP-induced activation of P2 × 7 receptor promoted the growth of tumors. Furthermore, P2 × 7 receptor activation down-regulated E-cadherin protein expression and up-regulated MMP-2 mRNA and concentration levels. Knocking down the expression of P2 × 7 receptor could significantly inhibit the increase in the expression of N-cadherin, Vimentin, Zeb1, and Snail induced by ATP. In addition, ATP time-dependently induced the activation of STAT3 via the P2 × 7 receptor, and the STAT3 pathway was required for the ATP-mediated invasion and migration. Our conclusion is that ATP-induced P2 × 7 receptor activation promotes the migration and invasion of colon cancer cells, possibly via the activation of STAT3 pathway. Therefore, the P2 × 7 receptor may be a potential target for the treatment of colon cancer.

## Introduction

Colon cancer is one of the common digestive system tumors, which is easy to invade and metastasize, and seriously affects human physical and mental health (Machala et al., 2019). The treatment of colon cancer mainly includes surgery, chemotherapy and radiotherapy, which increases the overall survival rate of patients with colon cancer, but invasion and metastasis are still the main cause of death of patients with colon cancer (Mehta and Patel, 2019). Therefore, it is of great significance to explore the relevant molecular basis and pathological mechanism affecting the pathogenesis of colon cancer, to inhibit the migration and metastasis of colon cancer, and to target the treatment of colon cancer. ATP is not only an important energy information substance, but also participates in the transmission of signals in the body, which plays an important role in regulating the life activities of cells including tumor cells (Ghosh et al., 2019; Nesci et al., 2019). Tumor cells can release large amounts of ATP into the microenvironment and play a role in regulating tumor progression by acting on other signaling molecule (such as P2Y and P2X receptors) (Lee et al., 2019). P2 × 7 receptor is an important member of the P2X family, which is a kind of ATP gated ion channel receptor. P2 × 7 receptor widely distributed in human tissue structure (Kan et al., 2019). P2 × 7 receptor is activated by ATP, which can open a pore for cations on the cell membrane (sodium and calcium ions influx, potassium ions outflow), allow molecules less than 900 Da to pass freely, affect the stability of the cell membrane skeleton and cell membrane fluidity, trigger intracellular molecular imbalance (Zhang et al., 2020). Certainly, P2 × 7 receptor plays an important regulatory role in tumorigenesis. Activation of P2 × 7 receptor can regulate the growth, proliferation, apoptosis, metastasis and invasion of tumor cells (Bergamin et al., 2019; Cao et al., 2019; Zhang et al., 2019a). Studies have shown that ATP-induced activation of P2 × 7 receptor can promote the migration and invasion of tumor cells (Jelassi et al., 2011; Adinolfi et al., 2012). Studies have also shown that reducing P2 × 7 receptor expression can inhibit the migration of breast cancer cells (Jelassi et al., 2013). According to reports on colon cancer research, some studies have shown that high expression of P2 × 7 receptor in the tissues of patients with colorectal cancer is closely related to the survival and prognosis of patients (Zhang et al., 2019b; Calik et al., 2020). Therefore, P2 × 7 receptor may become potential targets for cancer treatment. However, the role and molecular mechanism of P2 × 7 receptor in the migration and invasion of colon cancer cells have remained unclear. Therefore, the purpose of this study is to provide a new theoretical basis and data support for the treatment of colon cancer by exploring the effect and molecular mechanism of P2 × 7 receptor on the migration and invasion of colon cancer cells.

## Materials and Methods

### Cell Culture

LOVO and SW480 colon cancer cells were purchased from the Cell Bank of the Chinese Academy of Sciences (Shanghai, China), and cells were placed in a CO_2_ incubator (37°C 5%CO_2_) for cultivation. The next day, the culture dish was removed from the incubator, the culture medium was discarded in the culture dish, and washed it twice with sterile PBS. 2 ml of 0.25% trypsin was added for digestion for about 5 min. Subsequently, the digestion was terminated by adding an equal amount of DMEM medium containing 10% FBS (Doctor DE Biological, Wuhan, China), and the cell suspension was transferred to a centrifuge tube and centrifuged at 1200 rpm/min for 5 min. The supernatant was discarded in the centrifuge tube, 2 ml of medium was added to resuspend the cells, and the cells suspension was added to the new petri dish for cultivation. The liquid of the cells was changed every 2–3 days, and the growth of the cells was observed under the inverted microscope.

### Cell Scratch Assay

A Marker pen was used to draw 6 horizontal lines behind each 6-well plate (Doctor DE Biological, Wuhan, China). The horizontal lines should pass through each hole at 1 cm intervals. The cell suspension was added to each well (approximately 7 × 10^5^ cells) and placed the 6-well plate into CO_2_ incubator (37°C, 5% CO_2_) for cultivation. After observed that the cells covered the entire hole, scratches were made with the sterile 10 μl gun head, and then the cells was washed 3 times with sterile PBS. Added DMEM medium containing 10% FBS and putted it in a CO_2_ incubator for cultivation. Cells were treated with or without P2 × 7 receptor agonist (ATP or BzATP), P2 × 7 receptor antagonist A438079 or AZD9056, or ATP + P2 × 7 receptor antagonist (A438019 or AZD9056) [These drugs are purchased from Glpbio (California, United States)], observed and photographed under an inverted microscope at 0 and 24 h, respectively, and the percentage of wound healing between cell scratches was calculated.

### Transwell Invasion Assay

Analysis of colon cancer cell invasion ability by using 24-well Transwell chamber (Doctor DE Biological, Wuhan, China). The filter of the upper insert was coated with Matrigel before used and planted/200 μl serum-free cell suspension (1 × 10^5^ cells), and the lower insert was added with 200 μl of DMEM medium containing 10% fetal calf serum. After cells were treated with or without ATP (100, 200, and 300 μM), BzATP (10, 50, and 100 μM), A438079 (10 μM) or ATP + A436079 (10 μM) for 24 h, and the cells invaded by the Matrigel and flters were fixed with methanol for 30 min. Subsequently, the cells were stained with crystal violet and the cells were counted in five random fields under the microscope.

### Transwell Migration Assay

Analysis of colon cancer cell migration ability by using 24-well Transwell chamber (Doctor DE Biological, Wuhan, China). The upper inserts were seeded with 200 μl serum-free cell suspension (1 × 10^5^ cells), and the lower inserts were flled with DMEM medium supplemented with 10% FBS as a chemoattractant. After cells were treated with or without (100, 200, and 300 μM), BzATP (10, 50, and 100 μM), A438079 (10 μM) or ATP + A436079 (10 μM) for 24 h, and the migrated cells were fixed with methanol for 30 min. Subsequently, the cells were stained with crystal violet and the cells were counted in five random fields under the microscope.

### Western-Blotting

Total proteins were extracted from treated or untreated LOVO and SW480 cells. Separating glue and stacking glue separately were prepared, and then separating glue was added to the glass plate, about 3/5 of the glass plate. After separating glue was solidified, stacking glue was added and then the comb was inserted. After the stacking glue was solidified, the comb was pulled out, and then the gel was placed in an electrophoresis tank containing 1 × TEA electrophoresis solution (Boster Biological Technology, Ltd.). 3 μl marker (Boster Biological Technology, Ltd.) was added to the first well, and then 10 μl of samples were added to the subsequent wells in the order of the groups. The power supply was installed for electrophoresis about 90 min (voltage 120 V). After the electrophoresis was completed, the glass plate was opened and the gel was removed, and then placed in the transfer film frame. The appropriate nitrocellulose filter membranes (Boster Biological Technology, Ltd.) was putted on the surface of the gel and clamped the transfer film holder, and then placed into the electrophoresis tank containing transfer solution to transfer the membrane (current 260 mA, about 90 min). After the membrane transfer was completed, the membranes were removed and placed in 5% skimmed milk powder for 90 min. Subsequently, the membranes were placed in the primary rabbit P2 × 7 polyclonal antibody (1:1000, Millipore, Bedford, MA, United States); rabbit E-cadherin polyclonal antibody (1:1000, Millipore, Bedford, MA, United States); rabbit STAT3 polyclonal antibody (1:1000, Millipore, Bedford, MA, United States); p-STAT3 (1:1000, Millipore, Bedford, MA, United States), rabbit N-carherin polyclonal antibody (1:1000, Millipore, Bedford, MA, United States), rabbit Vinmentin polyclonal antibody (1:500, Millipore, Bedford, MA, United States), rabbit Zeb1 polyclonal antibody (1:1000, Millipore, Bedford, MA, United States), rabbit Snail polyclonal antibody (1:1000, Millipore, Bedford, MA, United States), or rabbit β-actin polyclonal antibodies (1:1000, Boster Biological Technology, Ltd.) and incubated overnight in a 4°C refrigerator. The next day, the membranes were removed and washed three times with TBST for 10 min each time. Then, the membranes placed in goat anti-rabbit secondary antibody (1: 3000, Boster Biological Technology, Ltd.) and incubated for 90 min, and then washed three times with TBST for 10 min each time. Subsequently, the chemiluminescence reaction was carried out. Grayscale analysis of the protein bands were carried out by using Image Pro Plus version 6.0 image analysis software. The relative band strength of the target protein was normalized with internal parameters (β-actin).

### RT-PCR

Total RNA were extracted from treated or untreated LOVO and SW480 cells, and reversed transcription into cDNA. Subsequently, the cDNA was further amplified (according to the instructions of Afnity Company PCR reaction reagents (Afnity company, United States) (reaction conditions: 94°C 5 min, 94°C 35 s, 56°C 30 s, 72°C 30 s, 72°C 10 min, 30 reaction cycles), the total system of P2 × 7 receptor was 20 μl. 1% agarose gel was made (weighed 0.6 g of agarose powder (Doctor DE Biological, Wuhan, China) and added 60 ml of 1XTAE), and then putted in the microwave oven and dissolved quickly. 2 μl of EB staining solution (Transgen, Beijing, China) was added and mixed, and then poured into the plate and inserted the comb. After the agarose gel was solidified, pulled out the comb, removed the gel and placed into the electrophoresis tank containing 1XTAE electrophoresis solution. 3 μl of marker (Doctor DE Biological, Wuhan, China) was added to the first well, and 10 μl of samples were added to the subsequent wells in the order of the groups. The power supply was installed and performed electrophoresis for about 40 min (voltage 120 V, current 140 mA). After the electrophoresis was completed, the gel was putted into the gel imaging system (Thermo Fisher Scientific, Madison, WI, United States) for observation and photography. The relative band strength of the target was normalized with internal parameters (β-actin).

P2 × 7 receptor primers:

Upstream: 5′-CTTCGGCGTGCGTTTTG-3′

Downstream: 5′-AGGACAGGGTGGATCCAATG-3′

β-actin primers:

Upstream: 5′-TAAAGA CCTCTATGCCAACACAGT-3′

Downstream: 5′-CACGATGGAGGGGCCGGACTCATC-3′

### RT-qPCR

Total RNA was extracted from treated or untreated LOVO and SW380 cells, and then reversed transcription into cDNA by using TagMan mRNA Reverse Transcription Kit (Doctor DE Biological, Wuhan, China) for PCR amplification (reverse transcription response system: 16°C 30 min, 42°C 30 min, 85°C 5 min). Subsequently, cDNA as a template, and Takara qPCR Kit (Wuhan Boshide Company, Wuhan, China Doctor DE Biological, Wuhan, China) was used to configure a 20 μl system for PCR reaction (reaction conditions: 95°C 30 s, 95°C 35 s, 60°C 1 min, 95°C 15 s, 40 reaction cycles). Each sample is set with three multiple wells, and β-actin was used as internal control.

MMP-2 primers:

Upstream: 5′-CAGGACATTGTCTTTGATGGCATCGC-3′

Downstream: 5′-TGAAGAAGTAGCTATGACCACCGCC-3′

N-cadherin primers:

Upstream: 5′-TGTTGCTGCAGAAAACCAAG-3′

Downstream: 5′-TTTCACAAGTCTCGGCCTCT-3′

Vimentin primers:

Upstream: 5′-GAGAACTTTGCCGTTGAAGC-3′

Downstream: 5′-GCTTCCTGTAGGTGGCAATC-3′

Zeb1 primers:

Upstream: 5′-GCACAACCAAGTGCAGAAGA-3′

Downstream: 5′-CATTTGCAGATTGAGGCTGA-3′

Snail primers:

Upstream: 5′-TTCTTCTGCGCTACTGCTGCG-3′

Downstream: 5′-AGAAGGAGAGGTATGGACGGG-3′

### Small Interference RNA Transfection

P2 × 7 siRNA (siP2 × 7) (5-CCGAGAAACAGGCGAUAAU-3) and a scramble sequence not targeting any known gene was used as a control siRNA (siCtrl) (Doctor DE Biological, Wuhan, China). LOVO and SW480 colon cancer cells were seeded in 24-well plates at a density of 1 × 10^5^/ml. After 6 h, the cells were transfected with siP2 × 7 and siCtrl by using Lipofectamine 3000. After 48 h of transfection, the real-time PCR was used to detect the gene knockout efficiency.

### Enzyme-Linked Immunosorbent Assay

After LOVO and SW480 cells were treated with or without ATP or BzATP, the cell culture supernatant was collected and centrifuged at 1200 rpm/min for 5 min. The concentration levels of MMP-2 were measured by using an Enzyme-Linked Immunosorbent Assay (ELISA) kit (Doctor DE Biological, Wuhan, China). Subsequently, the test was carried out according to the instructions of the ELISA kit.

### Electrophysiological Recording

Whole-cell patch clamp recording used CEZ2400 magnifier (BioLogic Science Instruments, Claix, France), glass microelectrode container was filled with intracellular fluid to drive out excess bubbles, and adjusted the pH to 7.2. Adjusted phase boundary potential compensation, potential resistance was 2–4 Mn. Standard extracellular fluid was used to perfuse the cells, forming a high-resistance seal between the electrode and the cell, and sucking and breaking the cell membrane. Adjusted capacitance and series resistance compensation, and membrane potential was held at −60 mV. The membrane current was applied with low-pass filtering (1 KHz), and experiments were performed at room temperature (20∼25°C). Agonists ATP (100–300 μM) or BzATP (10, 50, and 100 μM) were externally applied using a micromanipulator fast-flow delivery system (BioLogic Science Instruments, Claix, France) for 10 s. Before recording the effect of ATP, antagonists A438079 and AZD9056 were added to the water bath for 2 min. The experimental results were recorded on the recorder. Origin 8.0 software was performed to plot concentration-responses curves (MicroCal Software Company).

### Fluo-4AM Assay

Fluorescence indicator Fluo-4AM (Doctor DE Biological, Wuhan, China) was used to measure the change of free internal calcium concentration. Taken out the 24-well culture plate, discarded the culture medium, and washed the cells three times with HBSS, 5 min each time. Added 300 μl I μM Fluo-4AM working solution and incubated at 37°C for 30 min, and then washed 3 times with HBSS. Then, cells were incubated for an additional 10 min in HBSS. A laser confocal microscope (Danmic Global, LLC, San Jose, CA, United States) was used to detect the Fluo-4 fluorescence signals, and averaged Fluo-4 fluorescence signal was obtained from three separate experiments.

### Cell Proliferation Assay

SW480 and LOVO cells were inoculated onto 24-well plates and treated with or without ATP (300 μM), BzATP (10 μM), A438079 (10 μM), AZD9056 (10 μM), ATP + A43B079 (10 μM) or ATP + AZD9056 (10 μM) for 24 h. When the cell density reaches 75–85%, added 200 μl of 10 μM EDU working solution to each well and incubated at 37°C for 2 h. After EDU labeling, cells were fixed with 4% paraformaldehyde at room temperature for 15 min. Then 50 μl 2 mg/ml glycine solution was added to react for 5 min, and 0.3% TriX-100 PBS was added to incubate for 10 min. Subsequently, Apollo and Hoechst staining solutions were added to each well for staining, observation and photograph were taken under a fluorescent confocal microscope (Danmic Global, LLC, San Jose, CA, United States). The target emits red light after successful dyeing.

### *In vivo* Experiment

All animal experiments were approved by the Institutional Animal Care of the Second Hospital Affiliated, Nanchang Chang University, China [(No. 2017[028])]. BALB/c nude mice were reared in an aseptic environment controlled by light and temperature in the laboratory. LOVO cells were collected and reconstituted in PBS (100 μl), and approximately 2 × 10^6^ cells. The cells were injected subcutaneously on both flank regions of 5-week-old male nude mice, and the xenografts were allowed to grow. When the diameter of the tumor was close to 5 mm, the mice were randomly divided into three groups with 8 mice in each group. The PBS (control), ATP (300 μM), or ATP + AZD9056 (10 μM) were injected to xenotransplant tissue at twice a week for 8 times. The tumor size was measured with Vernier calipers, induced tumor volume = [length *X* width^2^]/2 for about 1 month. Mice were monitored daily for signs of toxicity.

### Statistical Method

Test of significance was done with Student *t*-test using SigmaStat 3.0 software (MD, United States). Alternatively, When the homogeneity test of variance fails, Mann-Whitney rank sum test is used. *P*-values of less than 0.05 were considered to have significant statistical significance.

## Results

### Functional Characteristics of ATP-Gated Ion Channel P2 × 7 Receptor in Colon Cancer Cells

**FIGURE 1:**
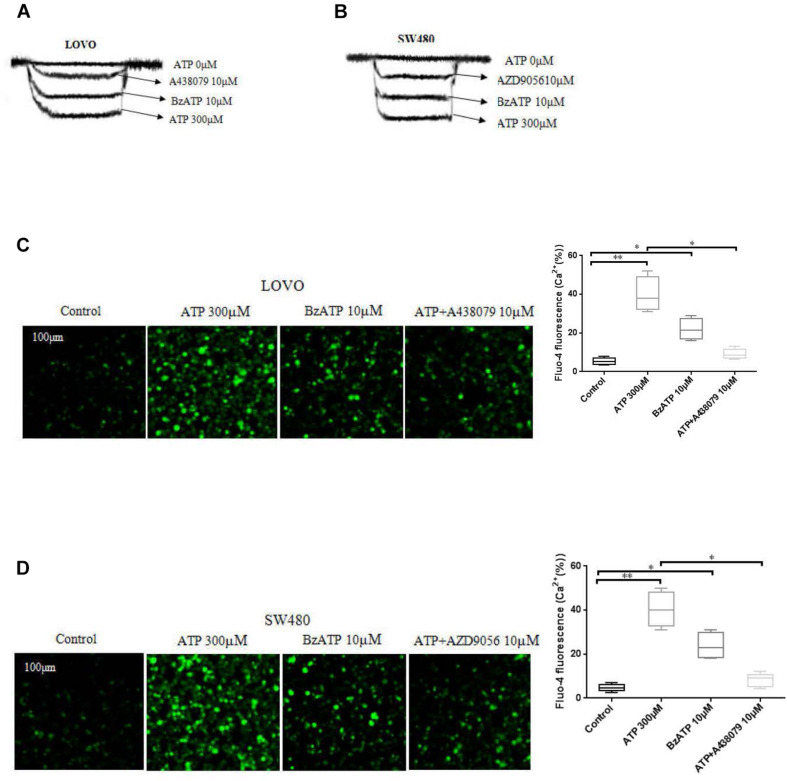
Characterization of ATP-gated P2 × 7 receptor functionality in colon cancer cell lines. **(A,B)**: LOVO and SW480 cells were treated with ATP (0 μM), ATP (300 μM), BzATP (10 μM), ATP + A438079 (10 μM) or ATP + AZD9056 (10 μM), inward current of electrophysiological recording. **(C,D)**: LOVO and SW480 cells were treated with ATP (300 μM), BzATP (10 μM), ATP + A438079 (10 μM) or ATP + AZD9056 (10 μM). Fluo-4AM fluorescence technology was performed to detect calcium changes in colon cancer cells. The data came from the average of three independent experiments. ^∗^*P* < 0.05, ^∗∗^*P* < 0.01.

To investigate the function of P2 × 7 receptor in colon cancer cells. We performed patch clamp and Fluo-4AM assays to evaluate the functional role of this receptor in LOVO and SW480 cells. The results showed that ATP (300 μM) significantly increased the inward current and intracellular calcium concentration. Similarly, ATP analog BzATP also obtained similar results. Moreover, before recording the effect of ATP, antagonists A438079 (10 μM) and AZD9056 (10 μM) were used and found that antagonists could significantly inhibit the inward current and intracellular calcium triggered by ATP (Figures 1A–D).

### Activation of P2 × 7 Receptor Promotes the Proliferation of Colon Cancer Cells

**FIGURE 2:**
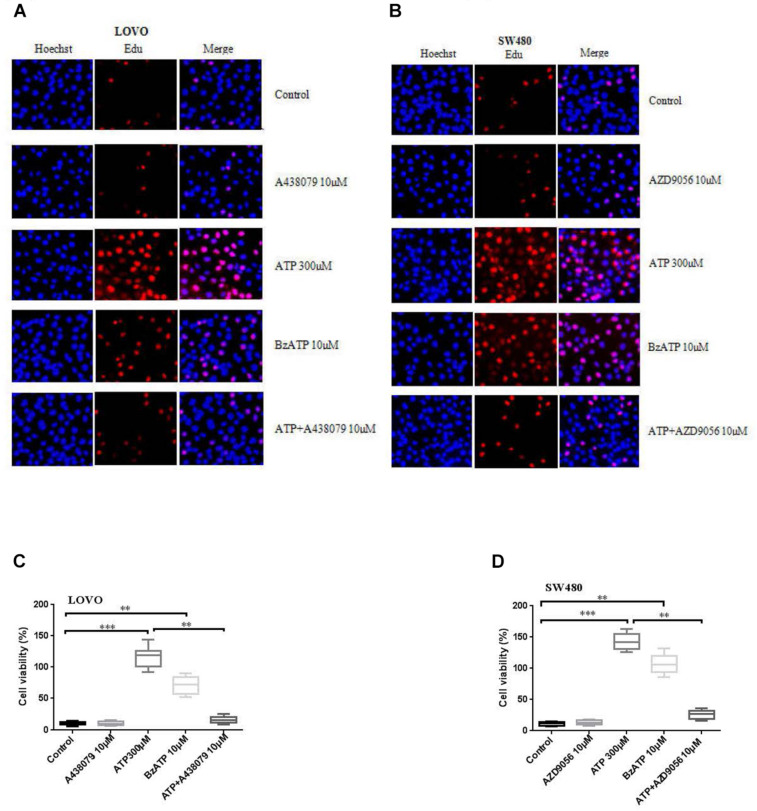
Activation of P2 × 7 receptor promoted the proliferation of colon cancer cells. **(A–D)**: LOVO and SW480 cells were treated with ATP (300 μM), BzATP (10 μM), A438079 (10 μM), AZD9056 (10 μM), ATP + A438079 (10 μM) or ATP + AZD9056 (10 μM) for 24 h. Edu assay was performed to detect the activity of colon cancer cells, bar = 100 μm, *n* = 3 independent experiments. ^∗∗^*P* < 0.05, ^∗∗∗^*P* < 0.01

To investigate the effect of P2 × 7R on the proliferation of colon cancer cells. LOVO and SW480 cells were treated or untreated with ATP (300 μM), BzATP (10 μM), A438079 (10 μM), AZD9056 (10 μM), ATP + A438079 (10 μM) or ATP + AZD9056 (10 μM) for 2 h. EDU assay was performed to detect the effect of P2 × 7R on the proliferation of colon cancer cells. The results showed that ATP significantly promoted the proliferation activities of colon cancer cells. Similarly, BzATP also significantly promoted colon cancer cell proliferation. Conversely, ATP + A438079 and ATP + AZD9056 obviously inhibited ATP-induced proliferation of LOVO and SW480 cells. However, in the absence of ATP, the use of antagonists had no obvious inhibitory effect on cell proliferation (Figures 2A–D).

### Extracellular ATP Promotes the Migration of Colon Cancer Cells

**FIGURE 3:**
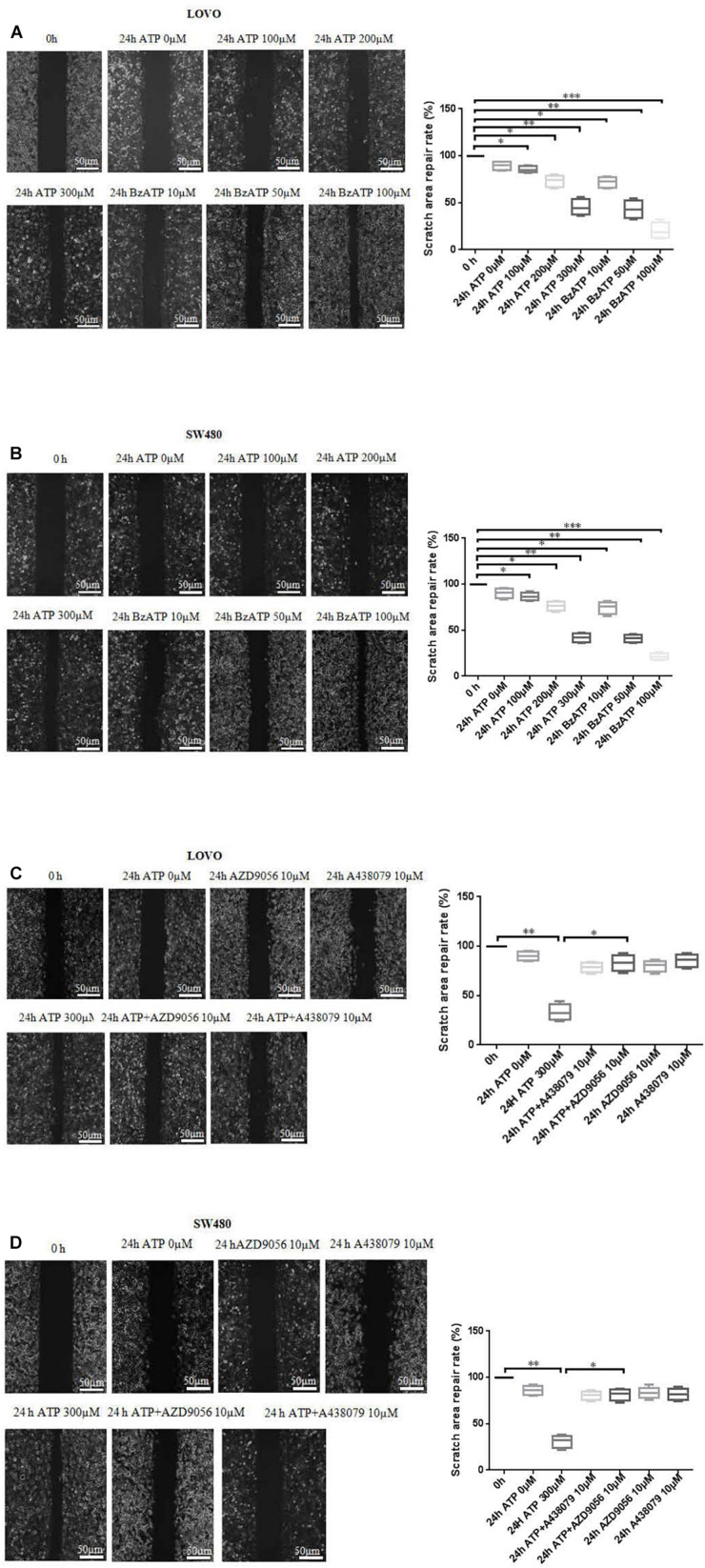
Cell scratch assay was performed to detect the role of extracellular ATP-medicated P2 × 7 receptor on the migration ability of colon cancer cells. **(A,B)**: LOVO and SW480 cells were treated with ATP (100–300 μM) or BzATP (10, 50, and 100 μM) for 24 h, and cell scratch assay was performed to detect the migration ability of colon cancer cells (bar = 50 μm). **(C,D)**: LOVO and SW480 cells were treated with ATP (300 μM), A438079 (10 μM), ATP + A438079 (10 μM), AZD9056 (10 μM) or ATP + AZD9056 (10 μM) for 24 h, and cell scratch assay was performed to detect the migration ability of colon cancer cells (bar = 50 μm) (*n* = 3 independent experiments). ^∗^*P* < 0.05, ^∗∗^ < 0.01.

To investigate the effect of P2 × 7 receptor agonists ATP, BzATP and antagonists A438079, AZD9056 on the migration of colon cancer cells. After the application of ATP or BzATP to LOVO and SW480 cells, the migration abilities of LOVO and SW480 cells was detected by cell scratch assay at 0 and 24 h, respectively. The results found that ATP (100–300 μM) dose-dependently increased the healing abilities of LOVO and SW480 cells. Similarly, ATP analog BzATP (10, 50, and 100 μM) also significantly promoted the healing ability of LOVO and SW480 cells in a dose-dependent manner (Figures 3A,B). These data indicate that extracellular ATP or BzATP can promote the migration of colon cancer cells.

To further determine the role of ATP-mediated P2 × 7 receptor activation in the migration of colon cancer cells. We used P2 × 7 receptor antagonists ATP + A438079 (10 μM) or ATP + AZD9056 (10 μM) to treat LOVO and SW480 cells and found that ATP-induced migration of LOVO and SW480 cells was significantly inhibited. However, in the absence of ATP induction, the inhibitory effect of antagonists on LOVO and SW480 cell migration were not obvious (Figures 3C,D). These data confirm that ATP-mediated P2 × 7 receptor activation promotes the migration of colon cancer cells.

### Effect of Extracellular ATP on the Invasion and Migration of Colon Cancer Cells

**FIGURE 4:**
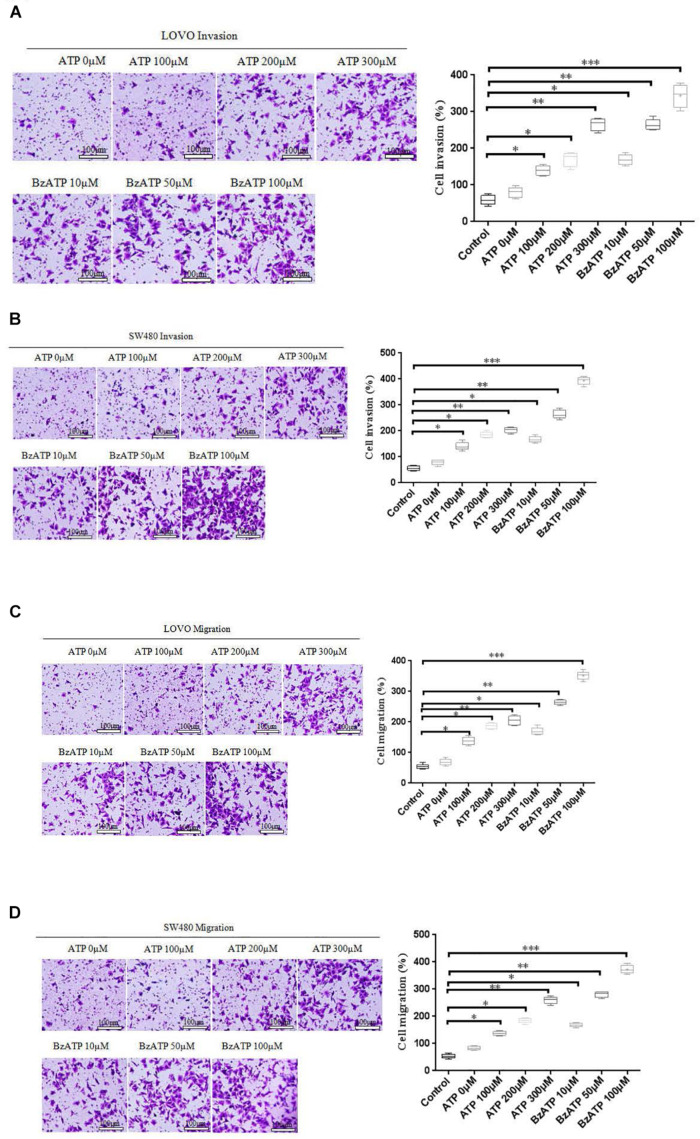
Effect of extracellular ATP or BzATP on the invasion and migration of colon cancer cells. LOVO and SW480 cells were stimulated with different concentrations of ATP (100–300 μM) or BzATP (10, 50, and 100 μM) for 24 h. **(A,B)**: Transwell invasion assay was performed to detect the effect of ATP or BzATP on the invasion abilities of colon cancer cells (bar = 100 μm). **(C,D)**: Transwell migration assay was performed to detect the effect of ATP or BzATP on the migration abilities of colon cancer cells (bar = 100 μm). *n* = 3 independent experiments. ^∗^*P* < 0.05, ^∗∗^*P* < 0.01, ^∗∗∗^ < 0.001.

To investigate the effect of extracellular ATP on the invasion and migration of colon cancer cells. We examined the effect of different concentrations of ATP (100, 200, and 300 μM) and BzATP (10, 50, and 100 μM) on the invasion and migration of colon cancer cells via Transwell invasion and migration assay. The results showed that, after 24 h of ATP-treated LOVO and SW480 cells, ATP produced a concentration-dependent increases in the migration and invasion capacities of LOVO and SW480 cells. Similarly, BzATP also significantly promoted the invasion and migration abilities of LOVO and SW480 cells (Figures 4A–D). These data once again show that extracellular ATP have a significant promotion effect on the invasion and migration of colon cancer cells.

### Effect of P2 × 7 Receptor Activation on the Invasion and Migration of Colon Cancer Cells

**FIGURE 5:**
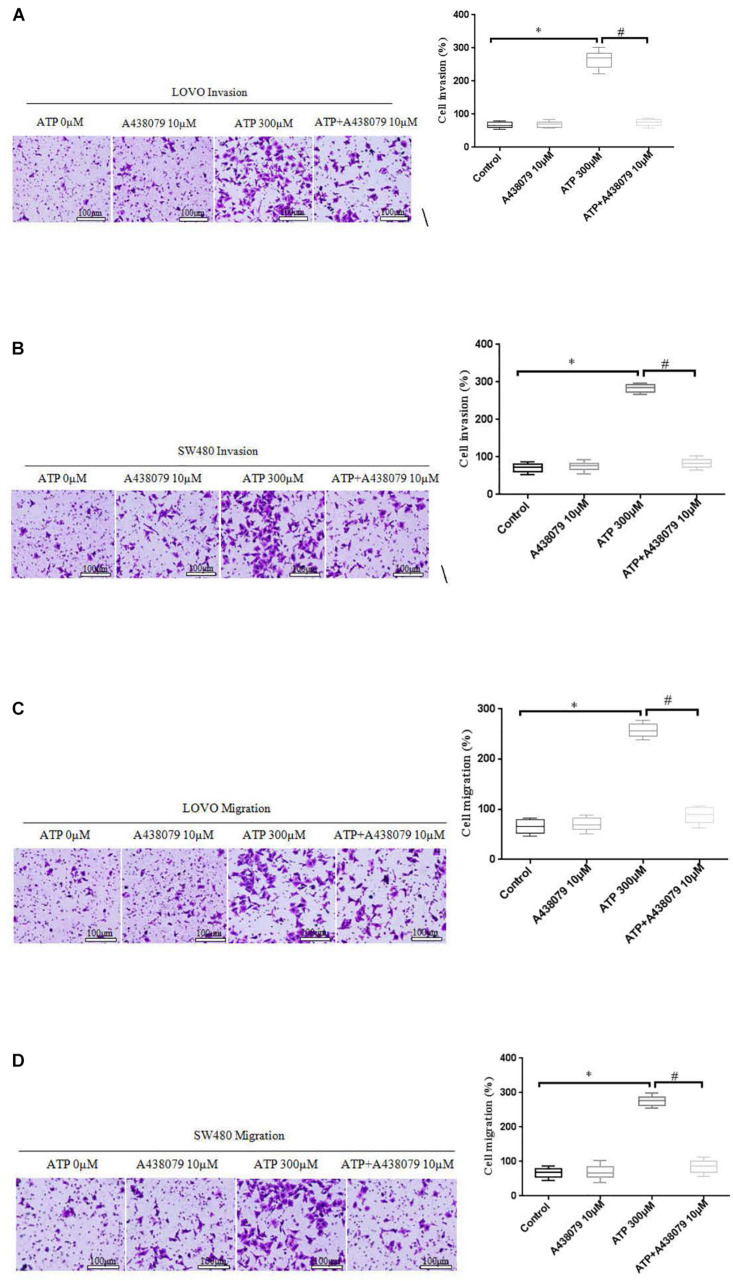
Role of P2 × 7 receptor in mediating the migration and invasion of colon cancer cells. **(A–D)**: LOVO and SW480 cells were treated with ATP (300 μM), A438079 (10 μM) or ATP + A438079 (10 μM) for 24 h. Effect of P2 × 7 receptor on the invasion and migration of LOVO and SW480 cells (bar = 100 μm). *n* = 3 independent experiments. ^∗^*P* < 0.05 ATP VS Control; ^#^*P* < 0.05 ATP + A438079 VS ATP.

To determine whether P2 × 7 receptor activation can promote the invasion and migration of colon cancer cells. On the basis of ATP (300 μM) or without ATP induced the invasion and migration of LOVO and SW480 cells, LOVO and SW480 cells were treated with or without P2 × 7 receptor antagonist A438079 (10 μM) for 24 h. It was found that A438079 significantly reduced the ability of ATP-induced invasion and migration of LOVO and SW480 cells. However, in the absence of ATP induction, the inhibitory effect of antagonists on LOVO and SW480 cell migration and invasion were not obvious (Figures 5A–D).

**FIGURE 6:**
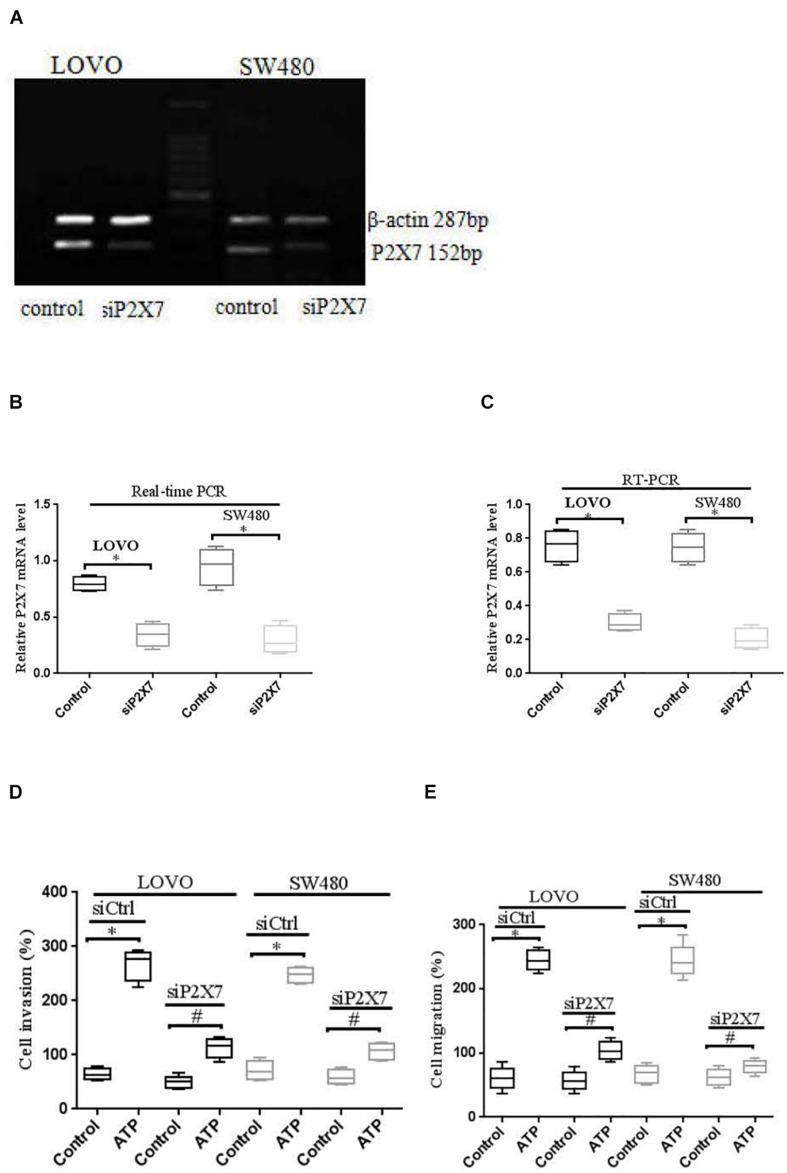
P2 × 7 receptor was involved in ATP-mediated the invasion and migration of colon cancer cells. **(A)**: RT-PCR was used to detect the expression of P2 × 7 receptor in LOVO and SW480 cells. **(B,C)**: LOVO and SW480 cells were transfected with control siRNA (siCtrl) or P2 × 7 siRNA (siP2 × 7) for 24 h, and knockdown efficiency was examined by real-time PCR and Western-blotting. ^∗^*P* < 0.05 siP2 × 7 VS Control. **(D,E)**: Effect of P2 × 7 receptor knockdown on ATP-induced the invasion and migration of LOVO and SW480 cells. *n* = 3 independent experiments. ^∗^*P* < 0.05 siCtrl ATP VS siCtrl control; ^#^*P* < 0.05 siP2 × 7 ATP VS siCtrl ATP.

Moreover, real time PCR and RT-PCR were performed to detect the expression level of P2 × 7 receptor in LOVO and SW480 cells, it was found that P2 × 7 receptor was highly expressed in LOVO and SW480 cells (Figures 6A–C). Therefore, we silenced P2 × 7 expression by siP2 × 7 transfection technology to investigate the effect of P2 × 7 knockdown on the invasion and migration of colon cancer cells, and knockdown efficiency was examined by real-time PCR and Western-blotting. It was found that knockdown of P2 × 7 receptor expression significantly reduced ATP-induced the invasion and migration of LOVO and SW480 cells (Figures 6D,E). These data indicate that activation of P2 × 7 receptor promotes the invasion and migration capabilities of colon cancer cells.

### ATP-Induced the Activation of P2 × 7 Receptor Affects the Expression the Expression of EMT-Related Genes

**FIGURE 7:**
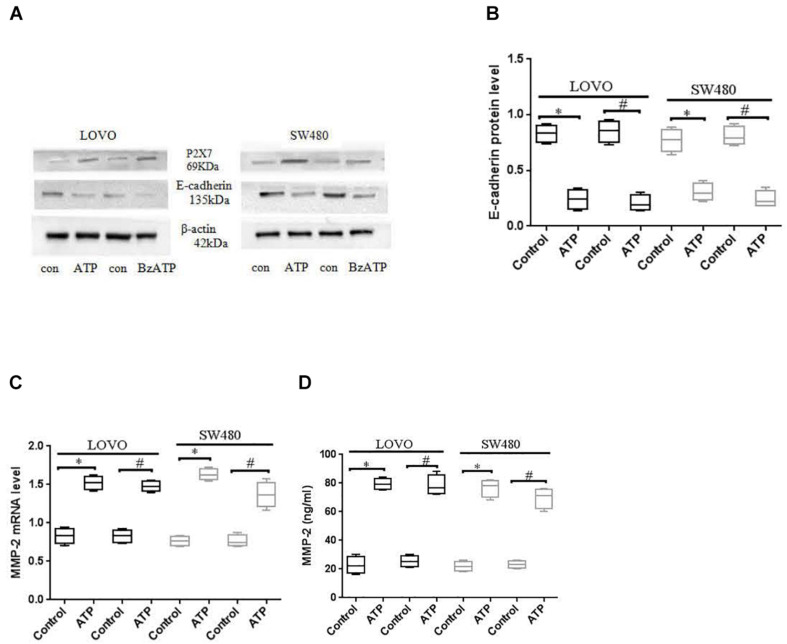
Effect of P2 × 7 receptor on the expression of E-cadherin and MMP-2. **(A,B)**: LOVO and SW480 cells were treated with ATP (300 μM) or BzATP (10 μM), Western-blotting was used to analysis the protein expression of E-cadherin. **(C,D)**: LOVO and SW480 cells were treated with ATP (300 μM) or BzATP (10 μM), real-time PCR and ELISA were used to analysis the expression level of MMP-2 mRNA and the concentration level of MMP-2. *n* = 3 independent experiments. ^∗^*P* < 0.05 ATP VS Control; ^#^*P* < 0.05 BzATP VS Control.

E-cadherin and MMP-2 are key factors in tumor migration and invasion (Padmanaban et al., 2019; Song et al., 2019). We analyzed the expression of E-cadherin and MMP-2 after LOVO and SW480 cells were treated with ATP (300 μM) or BzATP (10 μM). The results showed that ATP and BzATP significantly reduced the protein expression of E-cadherin in LOVO and SW480 cells (Figures 7A,B). Moreover, We analyzed the expression of MMP-2 mRNA by using real-time PCR in LOVO and SW480, ELISA was used to detent the concentration level of MMP-2 in LOVO and SW480 cell supernatant. The results found that ATP and BzATP significantly increased MMP-2 mRNA expression and concentration levels (Figures 7C,D).

**FIGURE 8:**
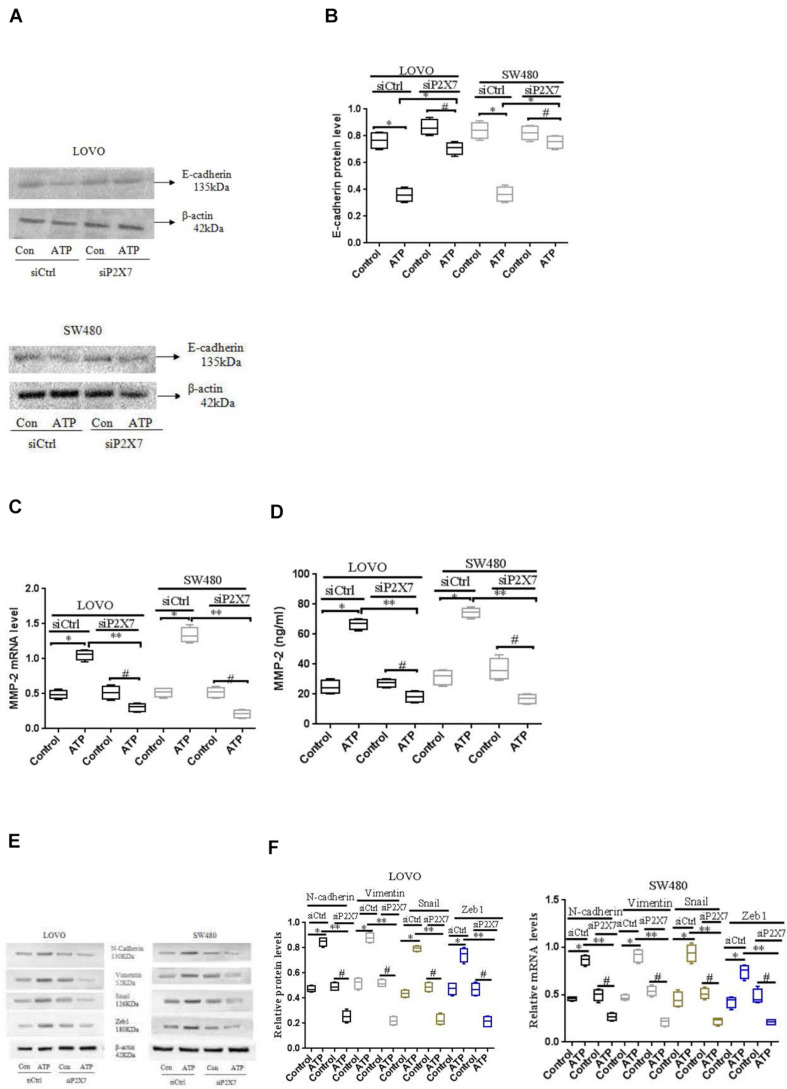
Effect of P2 × 7 receptor knockdown on the expression of E-cadherin, MMP-2, N-cadherin, Vementin, Zeb1 and Snail **(A,B)**: Western-blotting was used to analysis of the expression level of E-cadherin in siCtrl or siP2 × 7 LOVO and SW480 cells. **(C,D)**: Real-time PCR and ELISA were used to detect the expression of MMP-2 in siCtrl or siP2 × 7 LOVO and SW480 cells. **(E,F)**: Western-blotting and real-time PCR was performed to measure the expression of N-cadherin, Vimentin, Zeb1 and Snail preotein and mRNA in siCtrl or siP2 × 7 LOVO and SW480 cells. *n* = 3 independent experiments. ^∗^*P* < 0.05 siCtrl ATP VS siCtrl con; ^∗^*P* < 0.05 siCtrl ATP VS siCtrl con. ^∗∗^*P* < 0.05 siP2 × 7 ATP VS siCtrl ATP, ^#^*P* < 0.05 siP2 × 7 ATP VS siP2 × 7 con.

To further verify the above results, we used siCtrl/siP2 × 7 to transfect LOVO and SW480 cells, and found that the expression level of E-cadherin protein was increased, while the expression level of MMP-2 was reduced. In addition, in order to better illustrate that the P2 × 7 receptor is involved in the EMT process, we further measured the expression of other EMT-related genes. Western-blotting and real-time PCR were performed to measure the expression of N-cadherin, Vementin, Zeb1 and Snail in siCtrl or siP2 × 7 LOVO and SW480 cells. The results showed that knocking down the expression of P2 × 7 significantly reduced their expression levels (Figures 8A–F). These data further confirm that ATP-induced P2 × 7 receptor activation promotes the migration and invasion of colon cancer cells.

### Effect of P2 × 7 Receptor Activation on STAT3 Signaling in Colon Cancer Cells

**FIGURE 9:**
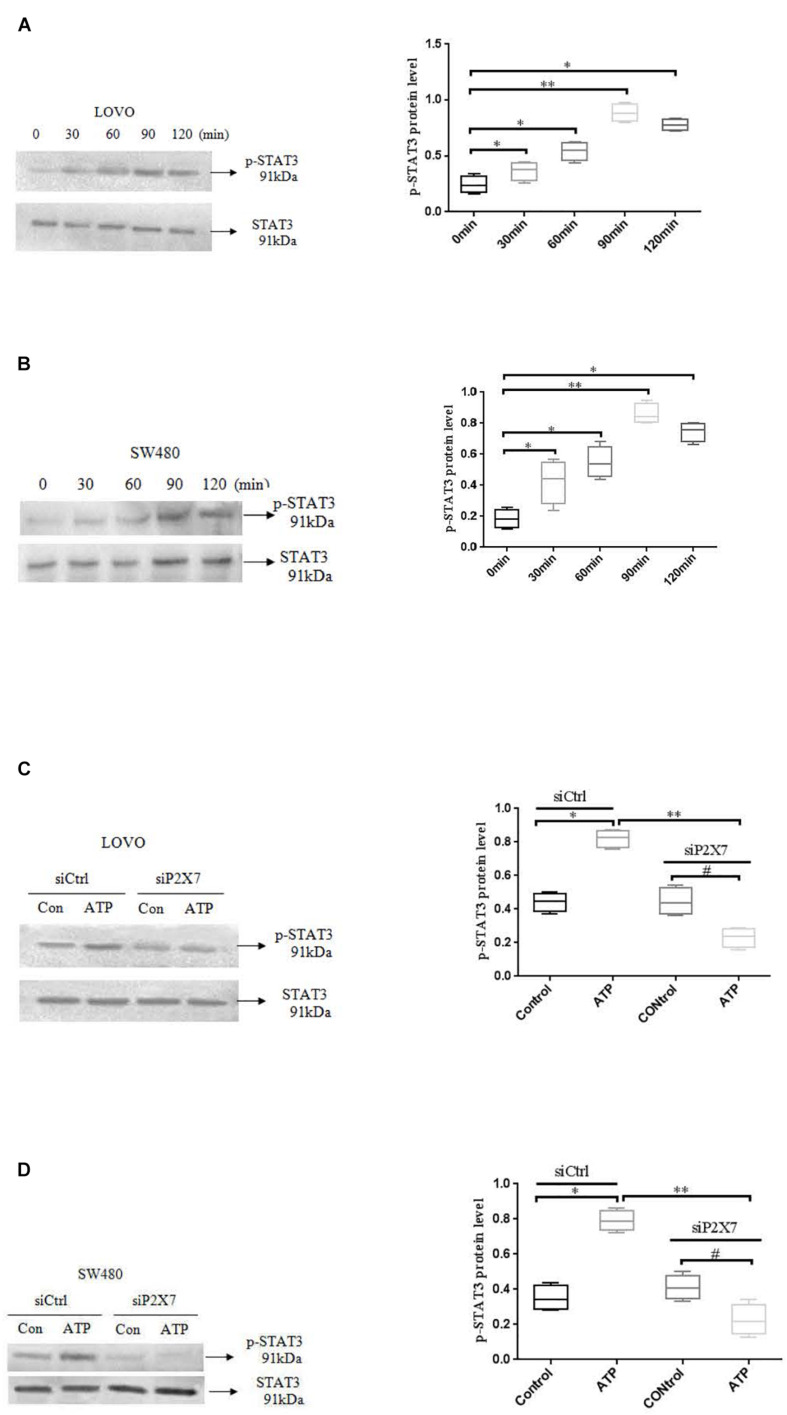
ATP-mediated activation of P2 × 7 receptor activated STAT3 signaling. **(A,B):** ATP (300 μM) stimulated LOVO and SW480 cells at 0, 30, 60, 90, and 120 min. Western-blotting was used to detect the expression level of p-STAT3. **(C,D):** LOVO and SW480 cells were treated with siCtrl or siP2 × 7 for 90 min. Western blotting was performed to detect the expression of p-STAT3 protein. *n* = 3 independent experiments. ^∗^*P* < 0.05 siCtrl ATP VS siCtrl con; ^∗∗^*P* < 0.05 siP2 × 7 ATP VS siCtrl ATP; ^#^*P* < 0.05 siP2 × 7 ATP VS siP2 × 7 con.

STAT3 pathway plays an important role in the development of tumor cells (Igelmann et al., 2019). While the role of P2 × 7 receptor-mediated STAT3 signaling in colon cancer has not been reported. Therefore, we investigated whether ATP-mediated P2 × 7 receptor activation has an effect on STAT3 signaling in colon cancer cells. Figures 9A,B showed that ATP (300 μM) increased the expression level of p-ATAT3 protein in LOVO and SW480 cells in a time-dependent manner (0, 30, 60, 90, 120 min), and the peak of p-STAT3 expression was at 90 min. Moreover, siP2 × 7 was used to knockdown of P2 × 7 expression, and found that p-STAT3 expression level was significantly reduced (Figures 9C,D). These data indicate that P2 × 7 receptor is involved in ATP-induced activation of STAT3 signaling in colon cancer cells.

### STAT3 Pathway Mediated the Role of P2 × 7 in the Migration and Invasion of Colon Cancer Cells

**FIGURE 10:**
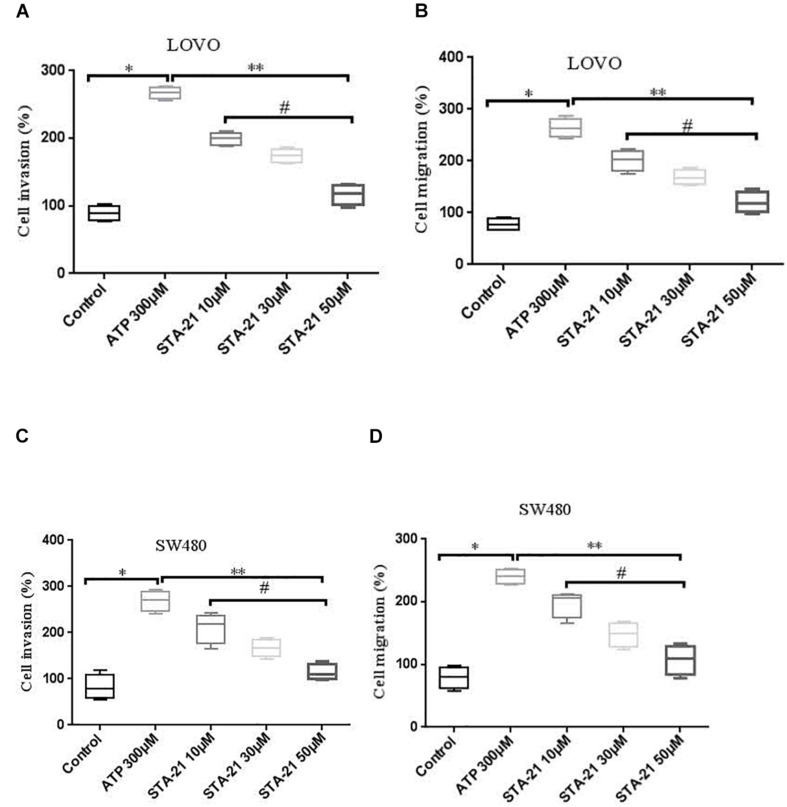
The role of P2 × 7 receptor mediated STAT3 in the migration and invasion of colon cancer cells. **(A,B)**: On the basis of ATP, LOVO cells were treated with STA-21 (10, 30, and 50 μM) (ATP + STA-21), respectively, and detected the migration and invasion abilities of LOVO cells by transwell assay. **(C,D)**: SW480 cells were treated with ATP + STA-21 (10, 30, and 50 μM), respectively, and detected the migration and invasion abilities of SW480 cells by transwell assay. *n* = 3 independent experiments. ^∗^*P* < 0.05 ATP VS Con; ^∗∗^*P* < 0.05 STA-21 VS ATP; ^#^*P* < 0.05 compared between STA-21.

To determine whether activation of P2 × 7 receptor promotes the invasion and migration of colon cancer cells via STAT3 signaling. We used STAT3 specific inhibitor STA-21 (ATP + STA-21 10, 30, and 50 μM) to treat LOVO and SW480 cells for 90 min. Transwell invasion and migration assay showed that STA-21 significantly inhibited ATP-induced migration and invasion of LOVO and SW480 cells, and inhibition of cell migration and invasion was concentration dependent on STA-21 (Figures 10A–D). These data determine that P2 × 7-mediated STAT3 signaling plays an important role in the invasion and migration of colon cancer cells.

### Effect of P2 × 7 Receptor-Mediated STAT3 Pathway on E-Cadherin and MMP-2 Expression

**FIGURE 11:**
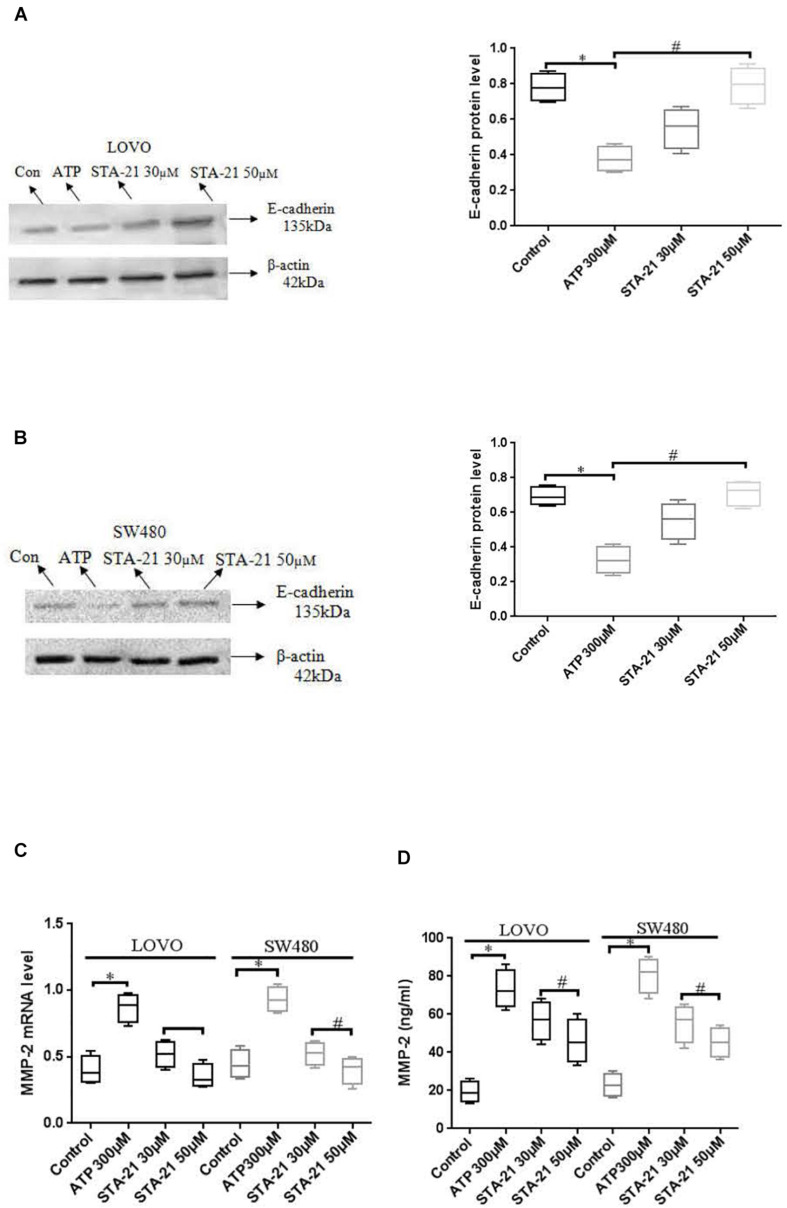
Effect of STAT3 pathway on the expression of E-cadherin and MMP-2 in colon cancer cells mediated by P2 × 7 receptor. LOVO and SW480 cells were treated with ATP (300 μM) or ATP + STA-21 (30 and 50 μM). **(A,B)**: Western-blotting was performed to analysis of protein levels of E-cadherin in LOVO and SW480 cells. (**C,D)**: Real-time PCR and ELISA were used to analysis of MMP-2 expression. *n* = 3 independent experiments. ^∗^*P* < 0.05 ATP VS Con; ^#^*P* < 0.05 STA-21 VS ATP.

To determine the effect of P2 × 7 receptor activation mediated STAT3 signaling on E-cadherin and MMP-2 expression. ATP (300 μM) or ATP + STAT3 inhibitor STA-21 (30 and 50 μM) was used to treat LOVO and SW480 cells. Figures 11A,B showed that ATP significantly reduced the expression level of E-cadherin protein, while STA-21 inhibited the ATP-induced down-regulation of E-cadherin protein expression. Moreover, real-time PCR and ELISA assay showed that ATP increased the expression level of MMP-2, while STA-21 inhibited ATP-induced up-regulation of MMP-2 expression (Figures 11C,D). These data indicate that activation of P2 × 7 receptor stimulates the STAT3 pathway and regulates MMP-2 and E-cadherin expression.

### Activation of P2 × 7 Receptor Promoted the Growth of Tumors *in vivo*

**FIGURE 12:**
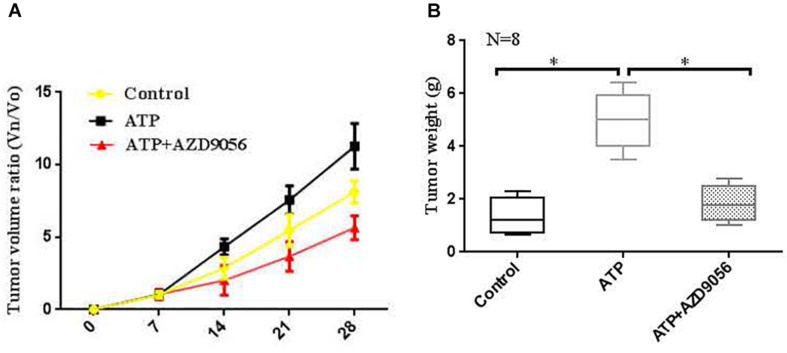
Activation of P2 × 7R promoted the growth of tumors *in vivo*. **(A,B)**: When the diameter of the tumor was close to 5 mm, the mice were randomly divided into 3 groups with 8 mice in each group. PBS (control group), ATP (300 μM), or ATP + AZD9056 (10 μM) were applied to induced the growth of tumor twice a week. The tumor growth was observed for 1 month, and the tumor volume and weight were measured. *N* = 8, ^∗^*P* < 0.05, ^∗∗^*P* < 0.01.

*In vivo* proliferative activity of P2 × 7 receptor on colon cancer was measured, and a subcutaneous xenograft model was created in nude mice. When the diameter of the tumor was close to 5 mm. The PBS (control), ATP (300 μM), or ATP + AZD9056 (10 μM) were injected to xenotransplant tissue at twice a week for 8 times. The volume of induced tumors was analyzed after the injection of PBS (control), ATP (300 μM) or ATP + AZD9056 (10 μM), induced tumor volume was measured every week. It was found that the tumor cells in the primary site invaded the surrounding tissues and found distant metastasis. especially in the ATP treatment group, which promoted the invasion and metastasis of tumor cells. Moreover, ATP treatment group increased the growth and weight of tumors, while ATP + AZD9056 group inhibited ATP-induced tumor growth and weight, and reduced the local invasion and distant metastasis of tumor cells (*P* < 0.05, *n* = 8) (Figures 12A,B). These data indicate that P2 × 7 receptor activation promotes the growth of colon cancer *in vivo*.

## Discussion

The process of migration and invasion of tumor cells is accompanied by a series of specific molecular events, such as immune cells, inflammation-related factors, and ATP (Morera et al., 2017; Zhang et al., 2019c). After the body is subjected to physiological and chemical stimulation (such as mechanical injury, inflammatory stimulation, hypoxia, acidosis, and tumor invasion), a large amount of intracellular ATP is released into the extracellular matrix and participates in cell biological activities (such as the survival, adhesion, proliferation, differentiation, and migration of cells) (Lertsuwan and Ruchirawat, 2017; Meurer et al., 2017). ATP plays an important regulatory role in the development of tumor cells and has been recognized by different studies (Jones et al., 2019; Kumari et al., 2019). High concentration of ATP in the tumor microenvironment can interact with other related molecules to play a role in regulating tumor progression. Previously, studies have found that extracellular ATP stimulation slows the growth of tumor cells (Yang et al., 2014). Later research found that ATP promotes the proliferation, migration and invasion of tumor cells (Shi et al., 2013). In this study, we found that ATP and BzATP significantly increased the inward current and intracellular calcium concentration of LOVO and SW480 cells, while the use of antagonists A438079 and AZD9056 could reverse the above phenomenon. Moreover, ATP could induce the migration and invasion of colon cancer cells in a concentration-dependent manner. Furthermore, BzATP could also promote the migration and invasion of colon cancer cells, confirming that ATP is involved in regulating the migration and invasion of colon cancer cells.

ATP can activate the P2 × 7 receptor and open the ion channels on the cell membrane, and affect the biological activities of cells (De Marchi et al., 2019). In the early stage, the function of P2 × 7 receptor was recognized mainly related to immune and inflammatory response. With the exploration of the function of P2 × 7 receptor, it was found that P2 × 7 receptor is closely related to the development of tumors (Zhang et al., 2020). Indeed, P2 × 7 receptor is expressed in a variety of tumor cells, such as lung cancer, prostate cancer, liver cancer, non-small cell cancer, and is closely related to the growth, proliferation, apoptosis, migration and invasion of tumors (Gilbert et al., 2019; Park et al., 2019). Studies have found that activation of P2 × 7 receptor activates PKC, ERK1/2, and JNK, and promotes the growth, proliferation and migration of pancreatic cancer cells (Choi et al., 2018). Activation of P2 × 7 receptor promotes the migration of lung cancer cells through regulation of actin cytoskeleton rearrangement (Takai et al., 2014). Studies have shown that the activation of P2 × 7 receptors induced by ATP and BzATP significantly promotes the migration and metastasis of pancreatic cancer cells, while P2 × 7 receptor antagonist (AZ10606120) can inhibit the proliferation of pancreatic cancer cells (Giannuzzo et al., 2016). Moreover, some *in vivo* studies have further shown that P2 × 7 receptor activation promotes tumor growth, while antagonizing or knocking down P2 × 7 receptor expression can inhibit tumor growth (Adinolfi et al., 2012, 2015; Brisson et al., 2020). However. studies have also shown that inactivation of P2 × 7 receptor can inhibit inflammatory bowel diseases, but it may increase the incidence of colitis-associated cancer (Hofman et al., 2015). In this study, it was found that P2 × 7 receptor was highly expressed in LOVO and SW480 cells. P2 × 7 receptor agonists ATP and BzATP could promote the invasion and migration of colon cancer cells, while P2 × 7 receptor antagonists A438079 and AZD9056 could inhibit ATP-induced the invasion and migration of colon cancer cells. Further transfection of LOVO and SW480 cells with siP2 × 7 inhibited the invasion and migration of colon cancer cells. Moreover, cell proliferation assay results found that ATP and BzATP significantly promoted the proliferation of colon cancer cells, while A438079 and AZD9056 could inhibit ATP-induced the proliferation of colon cancer cells. This result indicates that P2 × 7 receptor activation has a promoting effect on the growth of colon cancer. Furthermore, through *in vivo* models, it was found that P2 × 7 receptor activation promoted tumor growth. These results obtained in this study are consistent with the results of previous related studies, indicating that P2 × 7 receptor activation can promote tumor metastasis.

Epithelial-mesenchymal transition (EMT) is a necessary factor for the invasion and metastasis of tumor cells. ATP plays an important role in regulating tumor cell invasion and EMT (Yang et al., 2019). It has been found that extracellular ATP could modulate the MAPK signaling pathway to induce EMT and promote colon cancer cell metastasis (Huang et al., 2019). This study also found that ATP could induce EMT and promoted the metastasis of colon cancer cells. E-cadherin is an important EMT-related marker in tumors, which regulates cell-to-cell adhesion, and is usually weakly expressed in tumor cells (Menezes et al., 2019). Studies have shown that P2 × 7 receptor activation can promote the migration and metastasis of breast cancer cells by activating AKT signal and regulating the expression of MMP-13 and E-cadherin expression (Xia et al., 2015). *In vivo* and *in vitro* studies have shown that knocking down the expression of P2 × 7 receptor significantly inhibits the ATP or BzATP-driven expression changes of EMT-related genes Snail, E-cadherin, and MMP-3, and inhibits the migration and metastasis of prostate cancer cells (Qiu et al., 2014). In this study, we found that ATP and BzATP could down-regulate the expression of E-cadherin in the LOVO and SW480 cells. Transfection of LOVO and SW480 cells with siP2 × 7 confirmed that ATP reduced the expression of E-cadherin by activating P2 × 7 receptor. Moreover, MMP-2 plays an role in the migration and invasion of tumor cells (Ni et al., 2019). Different studies have shown that MMP-2 contributes to the migration and metastasis of colon cancer cells (Ko et al., 2019; Pietrangeli et al., 2019). Studies have shown that increased expression of P2 × 7 receptor can increase the expression of MMP-2 and MMP-9 through NF-kB, ERK1/2 and Akt signals, and promote the migration of prostate cancer and breast cancer cells (Tafani et al., 2011). However, other studies have found that P2 × 7 receptor had no significant effect on the expression of MMP-2, MMP-9, and EMT markers (Choi et al., 2018). Here, we found that ATP-induced P2 × 7 receptor activation increased the expression of MM9-2 in colon cancer cells. Furthermore, we also measured the expression of other EMT-related genes, and the results showed that knocking down the expression of P2 × 7 receptor significantly inhibited the increase in the expression of N-cadherin, Vimentin, Zeb1, and Snail induced by ATP. These results suggest that ATP-induced P2 × 7 receptor plays an important role in the EMT process. Our experimental results are consistent with the results obtained in some previous studies, indicating that P2 × 7 receptor can regulate the expression of MMP-2, E-cadherin, N-cadherin, Vimentin, Zeb1, and Snail, and affect the invasion and migration of colon cancer cells. However, some other studies have shown that ATP can affect the expression of EMT-related genes (such as MMP-3, E-cadherin, and claudin-1) through other P2 receptors (such as P2Y receptors) (Fang and Tian, 2017; Wang et al., 2017). Related studies have shown that ATP stimulation of P2Y2 receptors increases the expression of IL-8 and Snail, down-regulates the expression of E-cadherin and Claudin-1, and promotes the migration and metastasis of prostate cancer. While knocking down the expression of P2Y2 can inhibit prostate metastasis (Li et al., 2013). Other studies have also shown that the high expression of P2Y6 receptor increases the expression of vimentin in breast cancer cells and promotes the metastasis of breast cancer. It is further found that patients with high P2Y6 expression have a lower overall survival rate (Azimi et al., 2016). Therefore, our results only support that ATP-induced P2 × 7 receptor activation is involved in the progress of EMT, and more research data are needed in the future to determine the effect of P2 receptor on other EMT-related genes.

Many studies have reported that activation of P2 × 7 receptor can activate different intracellular signaling pathways (Koch-Nolte et al., 2019). Accordingly, activation of P2 × 7 receptor can also participate in tumorigenesis by activating different intracellular signals (such as NF-kB, ERK, AKT, and mTOR) (Qiu et al., 2014; Amoroso et al., 2015; Zhang et al., 2019a). STAT3 is an important signaling pathway in tumor development (Ashizawa et al., 2019). Studies have shown that TP53 missense mutation (mutp53) promotes the growth of colon cancer by driving Jak2/Stat3 signaling (Greten et al., 2018). However, there is no report on the effect of P2 × 7 receptor-mediated STAT3 signaling in colon cancer cells. Therefore, we investigated whether the P2 × 7 receptor can affect STAT3 signaling. In this study, we found that activation of P2 × 7 receptor up-regulated the expression of p-STAT3 in LOVO and SW480 cells and a dose-dependent manner with ATP concentration. While knocking down the expression of P2 × 7 receptor could reduce the phosphorylation level of STAT3. This may be due to knocking down the expression of the P2 × 7 receptor, resulting in restricted opening of ion channels on the cell membrane, thereby inhibiting STAT3 signaling. Moreover, blocking the STAT3 pathway could attenuate the effect of ATP-induced the invasion and migration of LOVO and SW480 cells. We further confirmed that blocking the STAT3 pathway could inhibit P2 × 7-mediated changes in the expression of E-cadherin and MMP-2. The results indicate that the activation of P2 × 7 receptor promotes the invasion and migration of colon cancer cells by activating the STAT3 signaling. However, the molecular mechanism that P2 × 7 receptor specifically affects STAT3 signaling requires more in-depth research and exploration. In addition, studies have found that ATP can directly pass through receptors other than P2 × 7 receptor (such as P2Y2 receptor) (Schulien et al., 2020), or indirectly through stimulation to induce the production of invasive molecules mediated by STAT3 (Yang et al., 2020), thereby promoting the diffusion of cancer cells. Therefore, based on these factors, our results only provide some data support for the role of ATP-mediated activation of P2 × 7 receptors in promoting the metastasis of colon cancer cells, but the molecular mechanism of ATP-mediated P2 × 7 receptor in tumor progression needs more in-depth exploration and mining.

From the experimental data obtained in this study, our conclude is that P2 × 7 receptor activation promotes the migration and invasion of colon cancer cells, while reducing P2 × 7 receptor expression can inhibit the migration and metastasis of colon cancer cell. The function of P2 × 7 receptor may be triggered by activating the STAT3 pathway. Therefore, P2 × 7 may be used as a new target for the treatment of colon cancer.

## Data Availability Statement

The raw data supporting the conclusions of this article will be made available by the authors, without undue reservation.

## Ethics Statement

The animal study was reviewed and approved by All animal experiments were approved by the Institutional Animal Care of the Second Hospital Affiliated, Nanchang University, China. Written informed consent was obtained from the owners for the participation of their animals in this study.

## Author Contributions

WZ carried out the study of the whole experiment and drafted the manuscript. CH and HL carried out western-blotting analysis and collected relevant data. ZZ carried out the design of the study and helped revise this manuscript. All authors read and approved the final manuscript.

## Conflict of Interest

The authors declare that the research was conducted in the absence of any commercial or financial relationships that could be construed as a potential conflict of interest.
